# Morbidity and Mortality After Second Hip Fracture With and Without Nursing Care Program

**DOI:** 10.7759/cureus.23373

**Published:** 2022-03-21

**Authors:** Konstantina Solou, Minos Tyllianakis, Antonis Kouzelis, John Lakoumentas, Andreas Panagopoulos

**Affiliations:** 1 Department of Orthopedics, School of Medicine, University of Patras, Patras, GRC; 2 Department of Medical Physics, School of Medicine, University of Patras, Patras, GRC

**Keywords:** nursing care, geriatric, morbidity, mortality, contralateral hip fracture, hip fractures

## Abstract

Background

Hip fractures are an increasingly common injury among older people who usually experience significantly worse mobility, independence in function, health, quality of life, and high rates of institutionalization. Studies have shown that only 40-60% of participants recover their pre-fracture level of mobility and ability to perform instrumental activities of daily living, while for those who are independent in self-care prior to the fracture, 20-60% still require assistance for various tasks one or two years after the fracture. As the cumulative incidence of a second hip fracture has been estimated to reach 8.4%, prevention of the second hip fracture is a major concern of the health system and the society focused mainly on lifestyle modifications, osteoporotic treatment, and fall-prevention strategies. The aim of the present study was to compare morbidity/mortality, functional results, and type of recovery between the first and second hip fractures in elderly patients.

Methods

Patients with a contralateral hip fracture were prospectively recruited during a three-year period (2016-2019). Level of independence, gait aids, type of rehabilitation, American Society of Anesthesiologists (ASA) physical status, Harris Hip Score (HHS), and Western Ontario and McMaster Universities Arthritis Index (WOMAC) scale were evaluated at admission for the second fracture and at the last reexamination.

Results

Twenty-seven out of 33 patients, aged 87.93±6.6, underwent surgery for contralateral hip fracture and followed up for 42.52±16.46 months; the mean interval between the two fractures was 39.63 months. The HHS averaged 86.19±12.18 and 59.01±32.83 and the WOMAC 86.37±12.09 and 68.22±26.18 before and after the second fracture, respectively. The mortality rate was 37.03%, 14.8±12.93 months after the second operation, with a significant relationship between the mortality time and the interval between fractures (p=0.028). Twelve and 14 of the patients received geriatric nursing care after the first and second fracture, respectively, without significant improvement in their functional results compared to home care. Mobility of nursing care patients after the second fracture was significantly improved (p=0.019).

Conclusions

Mortality is higher in the second year after the second hip fracture and strongly correlated with the time interval between fractures. A higher possibility to return in previous mobility status occurs after geriatric nursing care.

## Introduction

Fractures of the proximal femur, along with distal radius, proximal humerus, and vertebrae fractures, are associated with osteoporosis. The incidence of hip fractures fluctuates between 307 and 1269 per 100,000 in European countries. According to WHO, an increasing trend in the aging population is expected during the next decade; hence the prevalence of hip fractures will continue to grow globally [1]. Concerning morbidity and mortality of these fractures, studies have demonstrated that 25% of patients will return to their pre-fracture functional capacity, 50% will never recover to their pre-fracture state, and the rest 25% will die between the first six and 12 months [2-5]. As the natural history of osteoporosis is long, multiple fractures may occur to the same patient. Studies investigating the incidence and outcome of patients sustained a second hip fracture are mostly retrospective and do not include mobility and/or discharge destination data [2,6-8]. Various authors have analyzed outcomes in large samples and assessed the risk factors of a bilateral hip fracture, which consist namely age, sex, body mass index, osteoporosis evaluation, and prior functional status [9-13]. Long-term outcomes after surgically treated hip fractures, in terms of mortality and morbidity rate, are strongly associated with the rehabilitation pathway upon discharge from hospital and short-term independence after surgery, while pre-fracture residential status has been shown to be less significant [14]. Multidisciplinary models of rehabilitation after treatment of hip fragility fractures effectively improve mobility and autonomy status, with 32.1% of patients returning in full mobility or low impairment of mobility within the first year after such rehabilitation pathway [15].

Our hospital admits patients from larger rural areas who, upon discharge after a surgically treated hip fracture, return home to live under the care of relatives or neighbors or are transferred to nursing homes that provide no formal rehabilitation programs. Thus, in our study we mainly sought to assess morbidity and mortality in this highly fragile population.

## Materials and methods

Study design

We conducted a prospective study with blinded treatment and assessment to compare characteristics and functional outcomes of patients who sustained a second hip fracture. The ethics committee of Patras University Hospital has approved the study protocol, and each participant was informed in detail about the protocol.

Study population

Patients with a second hip fracture were recruited during a three-year period (from January 2016 to December 2019). During this period, a total of 587 patients with hip fractures (287 intertrochanteric and 300 femoral neck) caused by low-energy trauma were admitted to the hospital. Thirty-three among them sustained a second hip fracture; the demographic, clinical, and discharge data regarding their first hip fracture were retrospectively collected from our database. The surgical indications for the second fracture were peritrochanteric or femoral neck fracture. Patients were excluded if they were younger than 70 years, had a pathological fracture or a peri-implant fracture, or a subtrochanteric fracture. All these patients were followed-up after the second fracture at two weeks, one, three, six, 12 months postoperatively and yearly thereafter, until May 2021. For patients who could not visit the clinic, information was collected by phone from themselves, the caring persons, or both resources. We specifically sought to collect data about postoperative mobility, mortality, and functional activities. The final day of follow-up was determined to be either the date of the last follow-up or the date of death. During the study period, 27/33 patients were eligible and consented either directly or vicariously to enroll in this study.

Data retrieval

The retrieved records from our database included patient sex, date of birth, admission date, operation date, discharge date, diagnosis and preadmission and postadmission comorbidity diagnosis, place of accident, mechanism and date of hip fracture, type of the fracture, surgical treatment, length of stay, and time between the two fractures. We then asked the patients and their caring persons to retrieve information on mobility after the first fracture, anti-osteoporotic medication, and discharge destination. Mortality causes and death dates were also recorded. All these data were also prospectively collected after the second hip fracture.

Intervention

All patients underwent either intramedullary nail fixation or hemiarthroplasty or total hip arthroplasty, depending on their fracture type, comorbidities, and age. Postoperatively the patients and/or their caring people were given instructions on the physiotherapy protocol, tailored to each patient’s profile.

Outcomes

Clinical and radiographic assessments were conducted both prior to and after surgery. The preoperative raw data for each fracture included a full demographic profile, patient’s age, sex, and comorbidities classified according to the American Society of Anesthesiologists physical status classification system (ASA) [16]. For all patients with a second fracture, the Harris Hip Score (HHS) [17] and Western Ontario and McMaster Universities Arthritis Index (WOMAC) [18] questionnaires were completed for their status assessment at their admission for the second fracture and at the last follow-up. For both ASA and WOMAC, approval for use was sought. Patients were also asked whether they could walk independently, used walking aid (stick, crutches, walker), or were bedridden. Furthermore, their walking distance was classified as unlimited, 10km, 5km, in-home, or bedridden. Moreover, the discharge destination after each fracture was noted and categorized as house or nursing geriatric center, without formal rehabilitation facilities. Additionally, they were questioned if they were taking anti-osteoporotic treatment and which was the discharge destination after each operation for proximal hip fracture.

Statistical analysis

The aim of the analysis was to compare the patient’s characteristics after the first and after the second fragility hip fracture, as well as to relate their functional outcome and morbidity with discharge destination. All scale variables were initially examined for normality via the Shapiro-Wilk test for composite normality, and they were found to be not normal. Therefore, in what follows, non-parametric statistical tests are applied. Categorical-to-categorical variable dependencies were tested via Pearson’s chi-squared test of independence. Scale-to-categorical variable dependencies were estimated via Wilcoxon’s rank-sum test (equivalent to the Mann-Whitney test). Scale-to-scale variable correlations were assessed with Spearman’s Rho correlation test. Paired (i.e., before versus after) comparisons of scale scores (WOMAC and HHS) were examined with Wilcoxon’s signed-rank test. All tests were two-sided, while the statistical significance level was set at 5%. Survival analysis was used in order to study the duple dependent variable of time (follow-up time for alive patients or time to death for dead patients) plus event (a patient died or not); for that purpose, the Kaplan-Meier estimator was utilized. A variety of categorical or scale predictors were examined for the scenario to affect the survival with the log-rank test. The scale predictors (WOMAC and HHS scores) were discretized before, with the use of cut-offs that are traditionally applied. Finally, in order to model multivariate effects on the survival variable, Cox survival regression was performed (however, only demographic predictors were used because of the multicollinearity phenomenon and the lack of degrees of freedom issue). Statistical analysis was performed using the R statistical language and the RStudio software (The R Foundation, Vienna, Austria), both of which are open-source products.

## Results

The total number of patients admitted in our department for a low-energy hip fracture between January 2016 and December 2019 was 587. Thirty-three of them were surgically treated for a second hip fracture. Six patients (18%) could not be reached, and 27 patients completed the last follow-up (82%). Patients' demographic characteristics are presented in Table 1.

**Table 1:** Patients' demographic characteristics

Variables	Descriptive statistics
Age (years)	87.93±6.6 (72-100)
Gender	Females 23 (85.19%), males 4 (14.81%)
Follow-up time (months)	42.52±16.46 (18-66)
Fracture interval (months)	39.63±49.31 (3-240)
Mortality rate	10 (37.03%)
Life-time after second fracture (months)	14.8±12.93 (2-42)

Ten out of 27 (37.03%) had died by the time of the last follow-up. Thirteen patients were reached at the last follow-up, with an average follow-up time after the second hip fracture of 42.52 months (SD=±16.46). Two were males, and 25 were females. Their mean age at the first fracture was 84.3 (SD=±6.26) and at the second fracture was 87.93 (SD=±6.6). Eleven (40.7%) sustained a bilateral pertrochanteric hip fracture, six (22.22%) had bilateral femoral neck fracture, five (18.51%) had a femoral neck fracture following a pertrochanteric fracture, and five (18.51%) had a femoral neck fracture followed by a pertrochanteric fracture. In Table 2, the characteristics of the two groups of fractures are analyzed.

**Table 2:** Characteristics of the two groups of fractures ASA: American Society of Anesthesiologists physical status classification system; HHS: Harris Hip Score; WOMAC: Western Ontario and McMaster Universities Arthritis Index

		After the first fracture	After the second fracture
Intertrochanteric		16 (59.26)	16 (59.26%)
Femoral neck		11 (40.74%)	11 (40.74%)
ASA		2.56±0.51 (2-3)	3±0.5 (2-4)
HHS		86.19±12.18 (67.8-95.8)	59.01±32.83 (15.2-95.7)
WOMAC		86.37±12.09 (74.2-100)	68.22±26.18 (40.6-100)
Discharge destination	House	15 (55.55%)	19 (48.14%)
	Nursing geriatric center	12 (44.44%)	14 (51.85%)
Walking aid	Without walking aid	13 (48.15%)	6 (22.22%)
	Stick	9 (33.33%)	9 (33.33%)
	Walker	5 (18.52%)	8 (29.63%)
	Bedridden	0	4 (14.81%)
Walking distance	Unlimited	12 (44.44%)	4 (14.81%)
	10km	1 (3.7%)	1 (3.7%)
	5km	14 (51.85%)	5 (18.52%)
	In the house	0	13 (48.15%)
	Bedridden	0	4 (14.81%)

The interval between the two hip fractures was a minimum of three months and a maximum of 20 years (mean 39.63±49.31 months). Eight patients (29.62%) sustained the second hip fracture in the first year after the initial one, seven patients (25.9%) between the first and second year after the first fracture, while 12 patients (44.44%) had their second fracture in an interval of more than 24 months after their first hip fracture. The relationship between time interval and demographic and mobility factors after the first fracture is presented in Table 3 and demonstrates only a small but not statistically significant (p=0.066) correlation between time interval and mobility status after the first hip fracture.

**Table 3:** Correlations of the time interval between the two fractures and demographic and mobility factors ^1 ^Spearman's correlation test, ^2^ Wilcoxon's rank-sum test, ^3^ Kruskal-Wallis, ASA: American Society of Anesthesiologists physical status classification system; HHS: Harris Hip Score; WOMAC: Western Ontario and McMaster Universities Arthritis Index

Variable associated to time interval	P-value
Age^1^	0.552
Gender^2^	0.487
ASA score^2^	0.608
WOMAC after the first fracture^1^	0.188
HHS after the first fracture^1^	0.207
Discharge destination after the first fracture^2^	0.981
Mobility status after the first fracture^3^	0.066
Walking distance after the first fracture^3^	0.209

Comorbidities of patients at the first fracture as classified by ASA score were between 2 and 3 (mean 2.56±0.51), whereas at the second fracture mean ASA score was 3±0.5 (range 2-4). Twelve out of 27, and 14 out of 27 were discharged into a nursing geriatric center after the first and the second operation, respectively (p=0.589). Regarding functional outcomes, HHS was good, fluctuating between 67.8 and 95.8 (mean 86.19±12.18) after the first fracture, while the results were poor after the bilateral fracture, with a mean of 59.01±32.83 (range 15.2-95.7). Considering the second fracture, there is a statistically significant correlation in HHS between patients who returned home and the ones who continued their hospitalization in a geriatric nursing center, in favor of the latter (p=0.046), while there is no significant difference between the two groups after the first fracture (p=0.058). The WOMAC score was estimated by average 86.37±12.09 (range 74.2-100) and 68.22±26.18 (range 40.6-100) after the first and second hip fracture, respectively, without any correlation in the score between patients who followed different discharge destinations. There was an observed statistically significant decrease in the values of both scores, WOMAC and HHS, between the two fractures. The WOMAC score decreased on average 18.15±15.5 units, while the HHS score decreased on average 27.18±25.1 units (p<0.001 in either case). Both HHS and WOMAC, after the first and the second hip fracture, depend on mobility status and achieved walking distance, as expected.

After the first fracture, mobility was assisted with a stick in nine patients (33.33%), with walker in five (18.52%) patients and without any help in thirteen (48.15%) patients, of whom twelve (44.44%) walked an unlimited distance, one claimed walking up to 10km maximum, while fourteen (51.85%) could walk only 5km. After the second fracture, nine patients (33.33%) walked with a stick, eight were ambulating by using a walker, four (14.81%) were bedridden, and six (22.22%) could walk without any support. Four (14.81%) could walk unlimitedly/indefinitely, one could walk up to 10km, five (18.52%) could walk 5km, while thirteen (48.15%) could only be mobilized in their home, and four out of twenty-seven (14.81%) were bedridden. There was no statistically significant association between discharge destination and mobility status after the first hip fracture; however, after the bilateral hip fracture, there was a significant association (p=0.019) in favor of the nursing geriatric center. After the operation for the second hip fracture, nine out of twenty-seven (33.33%) patients returned to their previous mobility status, of whom 66.66%, after the second fracture fixation, were discharged to a geriatric nursing center. The correlations of functional scores with discharge destination are presented in Table 4.

**Table 4:** Identifying associations between discharge destination of patients in both fractures, and WOMAC and HHS scores and score differences between fractures, with the Wilcoxon's rank-sum test HHS: Harris Hip Score; WOMAC: Western Ontario and McMaster Universities Arthritis Index

Variable associated to discharge (first fracture)	home (n=15)	nursing house (n=12)	P-value
WOMAC	82.92±11.91	90.67±11.34	0.069
WOMAC difference	-23.13±14	-11.92±15.56	0.226
HHS	80.59±13.36	93.19±5.22	0.058
HHS difference	-31.8±24.61	-21.4±25.54	0.312
Variable associated to discharge (second fracture)	home (n=13)	nursing house (n=14)	P-value
WOMAC	57.21±24.7	78.44±23.97	0.107
WOMAC difference	-25.07±13.4	-11.72±14	0.110
HHS	45.97±32.36	71.12±29.34	0.046
HHS difference	-34.43±24.8	-20.44±24.31	0.148

**Figure 1:**
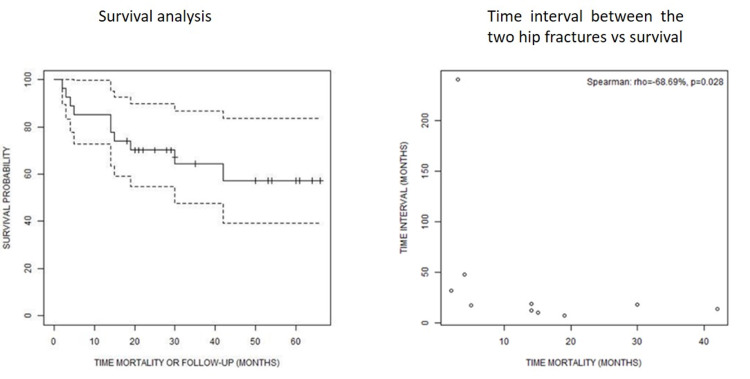
Survival rate 1a. Survival analysis, 1b. Time interval between the two hip fractures vs. survival

The mortality rate was 37.03%. Among 10 patients, who died in the meantime, the average time between operation for second fracture and mortality was 14.8±12.93 (range 2-42). Among them, four (40%) died during the first year after the second hip fracture, while four patients (40%) passed away 24 months after the second fragility hip fracture (Figure 1a). We found a negative, strong (-68.69%), and statistically significant (p=0.028) correlation between the time interval between the two fractures and the mortality time, including only the patients that deceased (Figure 1b). Survival analysis predicted resolution of 57.2% (standard error 11.1%, 95% confidence interval 39.2% - 83.5%), 42 months after the second fracture. Among potential associations between the survival variable and the rest of the factors, scale or categorical, only the mobility status after the second fracture was shown to affect survival (p=0.048). Cox regression modeling that used only the demographic variables revealed an association between the survival and the age in years (p=0.005), while the time interval between the two fractures, as well as gender and ASA, did not play a statistically significant role.

## Discussion

Epidemiological studies report increased osteoporotic fracture risk due to increasing life expectancy, and the absolute risk for a second osteoporotic-related fracture, in particular, is expected 28.6% [1]. In literature, contralateral osteoporotic hip fractures are reported approximately 8.54% (range 5-10%) [19-21]. Roux et al. [22], in the Fractos study (from the French national healthcare database), evaluated 356,895 patients hospitalized for severe osteoporotic fracture; in the 12 month-period following fracture, only 58,220 patients (16.7%) received a specific osteoporosis treatment, of whom 21,228 were previously treatment‐naïve. The 12‐month refracture rate and the all-cause mortality after the hip fracture have been estimated at 7.8% and 12.8%, respectively. The findings of this study emphasize the importance of better management of patients with severe fractures and of developing effective strategies to reduce fracture risk in patients with osteoporosis. The rehabilitation network of the public health system in Greece lacks organized geriatric teams and is not officially connected with hospitals, thus making rehabilitation care for elderly people that suffered a fragility fracture difficult and expensive. We sought to investigate the outcome of patients with a second hip fracture because this group is considered particularly fragile. In our institution, it constitutes 5.26% of patients who underwent surgery for an osteoporotic hip fracture.

The time interval between the two hip fractures is crucial. The average reported time in literature fluctuates between 31 months and 168 months, with 2-39% occurring during the first postoperative year [2,8-12,23]. In accordance with the presented data, the mean time interval between the first and second hip fracture of our group of patients was 39.63±49.31 months; however, 29.62% of the patients sustained the second fracture in the first year, and 25.9% of the elderly had their second fracture during the second postoperative year. There was no statistically significant correlation in the analysis of the relationship of the time interval with demographic and functional factors. A small association (p=0.066) was found between mobility status after the first fracture and time interval till the second fracture. Our high rates in the first year could be attributed to the lack of proper rehabilitation and the resultant prolonged impaired mobility. Moreover, usually, geriatric clinics fail to examine the elderly regularly; thus, their persistently impaired physical status, due to comorbidities, affects stability and independent ambulation. Studies have demonstrated that age and moderate functional status are causal factors for a second hip fracture [2,10]. Woo et al. [23], in their analysis, noticed that the second hip fracture in the trochanteric region occurs earlier than in the neck region (29.1 months vs. 37.4 months). Because of our small sample size, we could not investigate this relationship in our population.

Regarding mortality after a contralateral hip fracture, the recorded rate in published literature was 21% - 27.3% in the first year [1,2,9,10] and higher than the one noted at the first hip fracture, due to the longer bedridden state, poorer mental health, and longer time to reach the activity level. Smith et al. [24] performed a systematic review and meta-analysis, including 53 studies with 544,733 participants. Thirteen characteristics were identified as possible pre-operative indicators for mortality. In the following meta-analysis, the four key characteristics associated with the risk of mortality up to 12 months were abnormal ECG, cognitive impairment, age >85 years, and pre-fracture mobility. Other statistically significant pre-fracture predictors of increased mortality were male gender, being resident in a care institution, intra-capsular fracture type, high ASA grade, and a high Charlson comorbidity score on admission. A percentage of 37.03% of our patients died in a mean period of 14.8±12.93 months. Four and four out of 10 died in the first and second postoperative years, respectively. Investigating the correlations of survival with other demographic, functional, and mobility factors, we found a statistically significant correlation between mobility status after the contralateral fracture and survival (p=0.048), which is in accordance with the results of the studies mentioned above. We think that the fact that the mean patient’s age was 87.93, higher than the other studies, and the mean ASA score was 3±0.5 at the second fracture, compared to the 2 out of 4 of the rest of published studies could explain the higher mortality rates. Between our data, we have found that age is strongly negatively correlated to survival (p=0.017); however, there is no evidence that ASA score affects survival. Additionally, the time interval between the two fractures was strongly negatively correlated to survivorship (p=0.028). The predicted mortality risk after a second hip fracture was 57.2% (standard error 11.1%, 95% confidence interval 39.2% - 83.5%) after 42 months of the second hip fracture, according to our survival analysis.

A limited number of studies have explored the beneficial results of rehabilitation in elderly patients with hip fracture in terms of post-fracture function and ambulation [25-28]. Our findings concerning the association of mobility status after second fracture and mortality rate reinforce these conclusions. The postoperative independence at four months has been associated independently with the patient’s individual independence at hospital discharge and the presence of disorientation during hospitalization. Analyzing the correlation of the post-hospital care pathway to patients’ independence level, the intense rehabilitation program (three hours daily) presents satisfactory outcomes independently of the direct postoperative mobility status [14]. A study that compared the effects of rehabilitation between elderly who continued their hospitalization and patients who returned home early but followed a multifactorial rehabilitation program demonstrated that there is no difference in personal and instrumental activity of daily life (ADL) at three and 12 months with 40% of patients recovering to their pre-fracture ability [29]. None of our patients reported following an organized rehabilitation program at home or at a rehabilitation center after hospital discharge. Twelve (44.44%) and 14 (51.85%) patients after the first and the second hip fracture, respectively, were administered in a geriatric nursing center, but no official rehabilitation program was followed.

In addition to a potential phycological benefit, home discharge is preferable due to more sustainable costs for individuals and the healthcare system, thus rendering an informal caregiver preferable to a formal institution as a rehabilitation provider for an elderly patient after a hip fracture treatment. In our study, we found no statistically significant difference between home and geriatric nursing center in terms of mobility and functional scores, except that after the second hip fracture, the patients who were discharged in a nursing care center achieved significantly higher HHS score (71.12±29.34), compared to the ones discharged directly to their houses (45.97±32.36) (p=0.046). However, no correlation in the WOMAC score was noted. A feasibility study demonstrated that patients following the official rehabilitation program had better mobility in terms of walking aid and walking distance compared to those hosted postoperatively in nursing houses (p=0.02) at three months post-fracture. No difference was noted in motion and strength, while at 12 months follow up their mobility and quality of life remained higher in favor of rehabilitation protocol [30]. Our results showed that after the second hip fracture, 66.66% of those who returned to their previous mobility status were discharged to a geriatric nursing center without a formal rehabilitation program.

The high frequency of recurrence of osteoporotic fractures and the high rate of complications and morbidity between the elderly demonstrates the importance of a multidisciplinary team approach to achieve a well-structured anti-osteoporotic treatment protocol aiming at the development of higher compliance to therapy. According to new guidelines of the American Academy of Orthopaedic Surgeons (AAOS) and International Geriatric Fracture Society (IGFS), such a team should consist of orthopedic surgeons, emergency medicine physicians, geriatricians, anesthesiologists, cardiologists, nurses, physiatrists, and physiotherapists [31,32]. Systematic studies present improvement in clinical and cost-related outcomes and decreased hospitalization and mortality rate when patients refer to an orthogeriatric care model or a geriatrics-led care model [31]. Moreover, interdisciplinary teamwork between the health system, patients, and families should aim to prevent injuries such as falls and delirium, and provide global care to the elderly.

Some limitations of the present study should be noted. Our study is retrospective and partially based on information collected from patients or their carers; hence unavoidably biased. The small size of our sample is a possible bias as the correlations are not strong enough, even though the rate of patients who sustained a contralateral hip fracture in our hospital is in accordance with the published literature. Information on anti-osteoporotic medication was not fully collected for most of the patients, as they could not recall whether they were under treatment before the second fracture. Furthermore, poor compliance in anti-osteoporotic treatment was observed being a bias for further correlations.

## Conclusions

Studying a bilateral hip fracture population, we conclude that there is a statistically significant association in mobility status after second hip fracture in favor of nursing geriatric center hospitalization. Higher possibility to return in previous mobility status is associated with geriatric nursing center hospitalization. Mortality probability is higher in the second year postoperatively after the second hip fracture and strongly correlated with the time interval between fractures. A multidisciplinary team-based approach is essential for the prevention and management of osteoporotic fractures
